# Prostate cancer in Pennsylvania: The role of older age at diagnosis, aggressiveness, and environmental risk factors on treatment and mortality using data from the Pennsylvania Cancer Registry

**DOI:** 10.1002/cam4.3003

**Published:** 2020-03-25

**Authors:** Shirley M. Bluethmann, Ming Wang, Emily Wasserman, Chixiang Chen, Nicholas G. Zaorsky, Raymond J. Hohl, Alicia C. McDonald

**Affiliations:** ^1^ The Pennsylvania State University College of Medicine Hershey PA USA; ^2^ Penn State Cancer Institute Hershey PA USA

**Keywords:** aging, behavioral risk factors, geriatric oncology, healthy aging, prostate cancer survivorship

## Abstract

**Background:**

To assess: (a) cancer treatment in prostate cancer survivors (PCS) by age at diagnosis (ADx) and prostate cancer (PC) aggressiveness; (b) potential impact on PC mortality; and (c) these results in the context of environmental/behavioral risk factors on PCS in Pennsylvania.

**Methods:**

Prostate cancer survivors ages ≥40 years were identified from the 2004‐2014 Pennsylvania Cancer Registry (PCR). Demographic/clinical descriptors and PC treatment were extracted from PCR. Prostate cancer aggressiveness was defined by clinical/pathologic Gleason score and tumor stage. Logistic and Cox regression analyses tested associations between treatment received and PC‐specific mortality. County‐level data from the Pennsylvania BRFSS were used to estimate cancer‐related behavioral risk factors (eg, smoking, physical inactivity, fruit/vegetable consumption [FV], alcohol use) and used as covariates.

**Results:**

There were 90 694 PCS ages 40‐105 years (mean age = 66.19 years, SD = 9.25) included. Most were non‐Hispanic white men (83%). Prostate cancer survivors ≥75 years were least likely to receive any treatment but men ages 65‐74 were more likely to receive combined therapies (OR = 1.47; 95% CI 1.28, 1.69) vs PCS ages 40‐54 years, controlling for covariates. Prostate cancer survivors 55‐75+ with aggressive PC who received any treatment vs no definitive treatment had significantly reduced mortality. Men from counties with high obesity and smoking rates were significantly less likely to receive any treatment than men living in counties with lower rates of these risk factors. Prostate cancer survivors who lived in counties with high rates of physical inactivity and had high rates of sufficient FV consumption were slightly more likely to receive cancer treatment vs no definitive treatment compared to men who lived in counties with high rates of physical activity and lower FV consumption.

**Conclusions:**

We observed a general age‐related decline in receipt of treatment. Prostate cancer survivors ages ≥75 years were significantly less likely to get any cancer treatment compared to younger PCS. However, most men with more aggressive disease who received any treatment had greatly reduced PC mortality, regardless of age. Considering environmental/behavioral risk factors may attenuate PC risk and inform treatment options.

## Graphical abstract

We analyzed prostate cancer registry data for >90 000 men ages 40‐105 to understand age‐related associations with disease aggressiveness, treatment and potential role of environmental/behavioral risk factors in understanding likelihood of treatment recommendations and impact on mortality. Men older than 75 years were more likely to have aggressive disease but less likely to receive any treatment. The oldest men also had the greatest survival benefits from any treatment. We also provide insight on whether where you live impacts your treatment and ultimate survival.

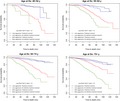

## 1 INTRODUCTION

Prostate cancer (PC) is the most commonly diagnosed non‐melanoma cancer in men in the United States,^1^ with more than 3 million US survivors (ie, individuals ever diagnosed with cancer). Most PC cases have a good prognosis even without treatment, but some cases can be aggressive and require multiple treatment modalities, including surgery (eg, prostatectomy)^2^, ^3^ and systemic therapy. Although PC mortality risk is lower than mortality from other cancer sites,^4^ it is recognized that the risk of PC death increases with advanced age. Currently, 70% of PC deaths occur in men ≥75 years.^5^, ^6^

With the aging of the US population, PC prevalence is expected to grow significantly, placing the oldest adults at the greatest risk.^7^ Pennsylvania has the fifth largest population of cancer survivors in the US.^8^ Pennsylvanian men may have unique risk factors from other men in the US, owing to geographic and sociodemographic characteristics such as diverse topography (ie, waterways and mountains) and a mix of rural and urban areas which may hinder healthcare access.^9^, ^10^ However, the population in Pennsylvania is not well studied relative to age or environmental (ie, non‐genetic) risk factors that may contribute to cancer disparities.^11^ Additionally, recent changes in recommendations for PC screening and treatment^12^ have complicated criteria by which treatment decisions for older PC survivors (PCS) are made.^13^, ^14^, ^15^ In particular, treatment patterns in older PCS (ie, ≥75 years) and effects on PC survival are still not well understood.^6^

The primary purpose of this study is to understand differences in diagnosis and treatment of PC by age, PC aggressiveness, and other sociodemographic and clinical characteristics at diagnosis among PCS in Pennsylvania. The aims were to: (a) assess cancer treatment patterns in PCS by age at diagnosis, sociodemographic factors, and PC aggressiveness; (b) understand the potential impact on PC‐specific mortality; and (c) to interpret these results in the context of county‐level environmental/behavioral factors associated with cancer risk.

## 2 METHODS

### 2.1 Data sources and analytical sample

#### 2.1.1 Pennsylvania Cancer Registry

Prostate cancer survivors ages ≥40 years were identified from the 2004‐2014 Pennsylvania Cancer Registry (PCR). For the analyses, age was categorized as follows: 40‐54, 55‐64, 65‐74, and 75+, based on observed distribution by age at diagnosis among PCR records of PCS. For disease aggressiveness, we defined the disease severity with two categories determined by Gleason score (GS) and tumor stage. Only cases defined by a GS ≥ 6 were included in the analysis, along with cases characterized by a tumor stage ≥T3 if the GS was missing or not documented. The pathologic GS and tumor stage were utilized whenever available (eg, at prostatectomy, surgery, autopsy), otherwise the clinical information was filled in (eg, biopsy, a transurethral resection of the prostate (TURP)). Depending upon the origin of the analytical GS, the corresponding GS pattern was extracted from the same origin. Using these analytical definitions, less aggressive was defined as GS 6‐7 (3 + 4) and tumor stage T1‐T2 with no distant metastasis, and more aggressive as GS ≥ 7(4 + 3) or tumor stage T3‐T4 excluding distant metastasis. This approach was informed by similar studies and standard staging criteria.^16^, ^17^, ^18^

#### 2.1.2 Pennsylvania behavioral risk factor surveillance system

Individual‐level behaviors and risk factors are not available in the PCR. To account for the potential impact of lifestyle and behavioral factors on PC risk related to health environment, we used county‐level estimates as proxies for this information, linking to individual‐level data by county at time of PC diagnosis. We requested aggregated 2011‐2014 prevalence estimates (%) by behavioral risk factor surveillance system (BRFSS) Region (groups of counties) for Pennsylvanian men age ≥40, to best overlap with our analytical PCR sample. Counties within the same BRFSS region were assigned the same values. The behaviors of interest were as follows: current smoking (defined as every/some days smoking AND having smoked ≥100 cigarettes in their lifetime); obesity (defined as BMI ≥ 30 kg/m^2^); physical inactivity (defined as reporting no leisure time physical activity in the past month); chronic use of alcohol/high risk for heavy drinking (defined as an average ≥2 drinks per day for the last 30 days); and fruit and vegetable (FV) intake (sufficient intake defined has having ≥5 servings of fruit and/or vegetables daily).^19^ Fruit and vegetable intake estimates were available from 2011 and 2013 survey years only.

### 2.2 Dependent variables of interest

The primary dependent variable of interest was PC treatment received, categorized based on common PC treatment modalities alone or in combination. These included: surgery only, radiation only, both surgery and radiation, and radiation and/or surgery with adjuvant therapy, including hormonal and/or chemotherapies (‘combined therapies’). We also captured cases in which ‘no definitive treatment’ was recorded. This category included individuals for whom no definitive treatment plan was found, which may include those patients were not eligible for radiation or chemo due to health concerns or preferences, or who receive palliative care only. Treatments received were determined by various PCR indicators, such as surgery type, radiation type, dates of administration, or dates of planned first course of treatment for the primary PC diagnosis. Any indication that a certain treatment was planned or administered generally overwrote any other unclear information recorded. Only when all information was missing or unknown was the treatment mode status considered ‘unknown.’ The primary surgery considered was radical prostatectomy. Any radiation treatment (eg, external beam radiation therapy or brachytherapy) was included for radiation. The primary hormonal treatment considered was androgen deprivation therapy. These treatment modalities are described as appropriate in international guidelines for PC management in older men.^20^, ^21^

For PC‐specific mortality, we used vitality data and cause of death records provided by the PCR, where cause of death is documented (code C61 or C619). Deaths due to other causes were treated as censored records.

### 2.3 Analysis

Multinomial logistic regression was used to assess the association between age at diagnosis and treatment received, adjusting for covariates such as race/ethnicity, insurance status, rurality, lymph node status, PC aggressiveness, serum PSA (natural log transformed), and behavioral risk factors from BRFSS (smoking, obesity, physical inactivity, chronic drinking, and FV intake). To further understand the potential survival benefit of receiving any treatment by age group,^22^, ^23^ hazard ratios (HR) were estimated using Cox proportional hazards (PH) regression for PC‐specific mortality, considering an interaction between PC aggressiveness and treatment status. For PC‐specific survival, Kaplan‐Meier estimates are calculated stratified by aggressiveness and treatment received status, and the comparison among survival curves are based on log‐rank tests. The PH assumption was checked based on graphical methods and statistical tests examining Schoenfeld residuals. All statistical tests were two sided using a significance level of *α* = 0.05. In cases in which some categories had small numbers of observations, the Hauck‐donner effect,^24^ which may inflate the likelihood of statistical significance, was observed and noted.

## 3 RESULTS

### 3.1 Characteristics of participants

Our analytic sample included 90 694 survivors ages 40‐105 years (mean age = 66.19 years, SD = 9.25) (see Table 1). The majority identified themselves as non‐Hispanic (NH) white (n = 75 161; 83%). Most were from urban settings (n = 69 866, 77%). In terms of clinical characteristics, most (n = 63 454, 70%) survivors were diagnosed between 55 and 74 years. More of the oldest survivors were diagnosed with aggressive disease. Approximately 30% (n = 9922) of men 65‐74 years had aggressive disease compared to 40% (n = 7039) of men ≥75 years. The most common cancer treatment modalities were surgery only (n = 31 077; 34%), radiation only (n = 20 266; 22%), combined therapies (radiation and/or surgery plus adjuvant therapies) (n = 15 624; 17%), and no definitive treatment (n = 20 138; 22%). However, this proportion varied across age groups (Table 1). In the oldest group, 25% (n = 4463) received all therapies combined or radiation only (n = 3962, 22%) and 44% (n = 7864) received no definitive treatment.

**TABLE 1:** Demographic and clinical characteristics of prostate cancer survivors by age at diagnosis

Demographic variables	Overall n = 90 694	40‐54 n = 9507 (10.48%)	55‐64 n = 30 318 (33.43%)	65‐74 n = 33 136 (36.54%)	75+ n = 17 733 (19.55%)	*P*‐value
Mean age in years (SD, range)	66.19 (9.25, 40‐105)	50.81 (2.95, 40‐54)	59.94 (2.78, 55‐64)	69.17 (2.87, 65‐74)	79.54 (4.03, 75‐105)	<.001
Race/Ethnicity n (%)						<.001
Hispanic (any race)	1246 (1.37)	185 (1.95)	468 (1.54)	443 (1.34)	150 (0.85)	
NH Black	9444 (10.41)	1600 (16.83)	3662 (12.08)	3013 (9.09)	1169 (6.59)	
NH White	75 161 (82.87)	7315 (76.94)	24 689 (81.43)	27 728 (83.68)	15 429 (87.01)	
Other	1103 (1.22)	108 (1.14)	360 (1.19)	447 (1.35)	188 (1.06)	
Unknown	3740 (4.12)	299 (3.15)	1139 (3.76)	1505 (4.54)	797 (4.49)	
Insurance status n (%)						<.001
Any private insurance/VA/TRICARE	33 503 (36.94)	6188 (65.09)	18 663 (61.56)	6589 (19.88)	2063 (11.63)	
Medicaid	1845 (2.03)	395 (4.15)	912 (3.01)	390 (1.18)	148 (0.83)	
Medicare only/Medicare plus private insurance	30 934 (34.11)	304 (3.20)	2299 (7.58)	17 989 (54.29)	10 342 (58.32)	
Medicare with Medicaid eligibility	924 (1.02)	100 (1.05)	216 (0.71)	435 (1.31)	173 (0.98)	
Other	6465 (7.13)	1005 (10.57)	3466 (11.43)	1379 (4.16)	615 (3.47)	
Uninsured	384 (0.42)	69 (0.73)	196 (0.65)	88 (0.27)	31 (0.17)	
Unknown	16 639 (18.35)	1446 (15.21)	4566 (15.06)	6266 (18.91)	4361 (24.59)	
Rurality n (%)						<.001
Urban cluster	6960 (7.67)	643 (6.76)	2041 (6.73)	2621 (7.91)	1655 (9.33)	
Rural	20 828 (22.97)	1953 (20.54)	6896 (22.75)	8073 (24.36)	3906 (22.03)	
Urbanized area	62 906 (69.36)	6911 (72.69)	21 381 (70.52)	22 442 (67.73)	12 172 (68.64)	
Prostate cancer characteristics						
Serum PSA score in ng/mL						<.001
*Mean (SD, range: 0.10‐98.00)*	10.30 (14.97)	8.53 (12.85)	8.95 (13.02)	10.22 (14.78)	14.14 (18.88)	
*Median (25th‐75th percentile)*	5.80 (4.40‐9.10)	5.10 (3.80‐7.60)	5.40 (4.20‐7.90)	6.00 (4.50‐9.00)	7.60 (5.10‐13.30)	
*Missing (n)*	11 640	943	3244	3812	3641	
Lymph node status n (%)						<.001
Negative	79 945 (88.15)	8768 (92.23)	27 740 (91.50)	29 373 (88.64)	14 064 (79.31)	
Positive (1 or more)	1248 (1.38)	197 (2.07)	474 (1.56)	400 (1.21)	177 (1.00)	
Unknown	9501 (10.48)	542 (5.70)	2104 (6.94)	3363 (10.15)	3492 (19.69)	
Prostate cancer aggressiveness n (%)						<.001
Less aggressive *Gleason score 6‐7 (3 + 4) and tumor stage of T1‐T2 and no distant metastasis*	56 121 (61.88)	6746 (70.96)	20 392 (67.26)	20 585 (62.12)	8398 (47.36)	
More aggressive^a^ *Gleason score ≥ 7 (4 + 3) to 10 or tumor stage of T3‐T4*	27 351 (30.16)	2287 (24.06)	8103 (26.73)	9922 (29.94)	7039 (39.69)	
Unknown	7222 (7.96)	474 (4.99)	1823 (6.01)	2629 (7.93)	2296 (12.95)	
Treatment summary n (%)						<.001
Surgery only	31 077 (34.27)	6418 (67.51)	15 791 (52.08)	8230 (24.84)	638 (3.60)	
Radiation only	20 266 (22.35)	1020 (10.73)	5735 (18.92)	9549 (28.82)	3962 (22.34)	
Radiation plus surgery	1387 (1.53)	277 (2.91)	715 (2.36)	370 (1.12)	25 (0.14)	
Radiation and/or surgery plus adjuvant therapy (HT and/or chemo)	15 624 (17.23)	647 (6.81)	3329 (10.98)	7185 (21.68)	4463 (25.17)	
No treatment	20 138 (22.20)	1035 (10.89)	4232 (13.96)	7007 (21.15)	7864 (44.35)	
Unknown	2202 (2.43)	110 (1.16)	516 (1.70)	795 (2.40)	781 (4.40)	

Note

All reported percentages are column percentages.

For Race/Ethnicity, if a case was documented as Black or White Race with Unknown Ethnicity, the Ethnicity was assumed to be Non‐Hispanic. If Race was Other/Unknown with Non‐Hispanic Ethnicity, categorized as Other. Only when both the Race and the Ethnicity items were both unknown was the final status treated as Unknown.

Rurality refers to the census tract definition where Urbanized areas are characterized by populations of ≥50 000; Urban clusters ≥2500 and <50 000; and Rural <2500.

Tumor stage was based on TNM 7.

Primary site surgery refers only to total organ resection (radical prostatectomy (NOS) not otherwise specified, total prostatectomy NOS, prostatectomy with resection in continuity with other organs, prostatectomy NOS).

Adjuvant therapy refers to either chemotherapy treatment and/or hormone therapy (HT) treatment.

No treatment includes those cases for which some form of adjuvant therapy was the only indicated treatment modality.

*P*‐values reflect the results from the one‐way ANOVA for continuous characteristics and the Chi‐square test for categorical characteristics to evaluate the association with age, with missing values and Unknown levels excluded.

Abbreviations: PCR, Pennsylvania Cancer Registry; PSA, prostate‐specific antigen (PCR documentation top‐coded at 98.0 and bottom‐coded at 0.1); SD, standard deviation.

^a^ Excludes distant metastasis.

### 3.2 Likelihood of receiving any cancer treatment modality

Using multinomial logistic regression to model the polytomous treatment modality response, we observed a general but significant increase in likelihood of receiving treatment by age at diagnosis, but treatment patterns shifted after age 75. Survivors diagnosed at ages 55‐64 years were 34% more likely (OR = 1.34:95% CI 1.2, 1.5) and survivors 65‐74 years were 60% more likely (OR = 1.6; CI 1.40, 1.8) to receive radiation only vs no definitive treatment than survivors 40‐54 years. Survivors 75+ years were 17% less likely (OR = 0.83; 95% CI 0.72‐0.96) to receive combined therapies (surgery ± radiation plus adjuvant therapy) than no definitive treatment compared to survivors 40‐55 years. Survivors 75+ were also 99% less likely to receive surgery only or radiation plus surgery only vs no definitive treatment compared to the youngest survivors ages 40‐54 years (Table 2).

**TABLE 2:** Adjusted multinomial logistic regression results for odds of receiving treatment modality

	Surgery only (vs no definitive treatment)	Radiation only (vs no definitive treatment)	Surgery and radiation only (vs no definitive treatment)	Radiation and/or surgery with adjuvant therapy (hormonal therapy and/or chemo) (vs no definitive treatment)
Age at Dx (y)				
40‐54 (REF)	—	—	—	—
55‐64	0.55 (0.49, 0.60)**	1.34 (1.19, 1.52)**	0.52 (0.43, 0.62)**	1.10 (0.96, 1.26)
65‐74	0.20 (0.18, 0.22)**	1.59 (1.40, 1.81)**	0.17 (0.14, 0.22)**	1.47 (1.28, 1.69)**
75+	0.01 (0.01, 0.01)^a^, **	0.80 (0.70, 0.91)**	0.01 (0.01, 0.02)^a^, **	0.83 (0.72, 0.96)*
Aggressiveness				
Less aggressive (REF)	—	—	—	—
More aggressive	2.39 (2.24, 2.54)**	0.67 (0.62, 0.71)**	14.55 (12.57, 16.83)**	4.15 (3.90, 4.41)**
Race/Ethnicity				
Non‐Hispanic White (REF)	—	—	—	—
Hispanic	0.87 (0.70, 1.08)	1.16 (0.93, 1.43)	0.87 (0.53, 1.43)	0.94 (0.74, 1.19)
Non‐Hispanic Black	0.53 (0.48, 0.57)**	0.91 (0.83, 0.99)*	0.63 (0.51, 0.77)**	0.78 (0.71, 0.86)*
Other	0.75 (0.60, 0.94)*	0.94 (0.75, 1.17)	0.76 (0.44, 1.30)	0.66 (0.51, 0.85)*
Insurance status				
Uninsured (REF)	—	—	—	—
Any private insurance/VA/Tricare	1.94 (1.40, 2.68)**	1.93 (1.31, 2.84)**	2.14 (1.00, 4.59)*	1.73 (1.18, 2.53)*
Medicaid	0.91 (0.64, 1.30)	1.66 (1.10, 2.51)*	1.49 (0.66, 3.38)	1.43 (0.95, 2.16)
Medicare only/Medicare plus private insurance	1.39 (1.00, 1.93)*	1.67 (1.14, 2.47)*	1.47 (0.68, 3.19)	1.54 (1.05, 2.26)*
Medicare with Medicaid eligibility	0.62 (0.42, 0.92)*	1.22 (0.79, 1.89)	0.91 (0.35, 2.40)	1.01 (0.65, 1.56)
Other	1.52 (1.09, 2.12)*	2.20 (1.48, 3.27)**	1.68 (0.76, 3.67)	1.59 (1.07, 2.35)*
Lymph node status				
Negative (REF)	—	—	—	—
Positive (1 or more)	0.31 (0.25, 0.38)**	0.10 (0.06, 0.15)^a^, **	0.60 (0.43, 0.83)*	1.07 (0.89, 1.28)
ln(serum PSA)^b^	0.68 (0.66, 0.70)**	0.81 (0.79, 0.84)**	0.87 (0.81, 0.94)**	1.13 (1.09, 1.16)**
Rurality				
Urban cluster (REF)	—	—	—	—
Rural	1.28 (1.16, 1.42)**	1.03 (0.93, 1.15)	1.16 (0.89, 1.52)	1.07 (0.96, 1.19)
Urbanized area	1.46 (1.32, 1.62)**	1.42 (1.29, 1.57)**	1.06 (0.81, 1.38)	1.11 (1.00, 1.24)*
Smoking	0.95 (0.94, 0.96)**	0.96 (0.95, 0.97)**	0.95 (0.93, 0.98)**	0.93 (0.92, 0.94)**
Obesity	0.96 (0.96, 0.97)**	0.96 (0.95, 0.97)**	0.92 (0.90, 0.94)^a^, **	0.97 (0.96, 0.98)**
Physical inactivity	1.02 (1.01, 1.04)**	1.02 (1.00, 1.03)*	1.04 (1.01, 1.08)*	1.07 (1.05, 1.08)**
Chronic drinking	1.00 (0.98, 1.02)	0.98 (0.96, 1.00)	0.98 (0.93, 1.03)	0.95 (0.93, 0.97)**
Daily fruits/vegetables	1.03 (1.02, 1.04)**	1.00 (0.99, 1.01)	1.04 (1.02, 1.07)**	1.02 (1.01, 1.03)**

Note

Cases with ‘Unknown’ or missing values for any of the covariates or the response were removed (n = 29 616 removed).

County‐level (BRFSS region) risk factors were assigned to PCR prostate cancer cases by county at the time of diagnosis, where counties within the same BRFSS region were linked with the same prevalence estimates as the region.

Odds ratio (95% confidence interval).

Abbreviation: BRFSS, behavioral risk factor surveillance system; PCR, Pennsylvania Cancer Registry; PSA, prostate‐specific antigen; REF, referent group.

^a^ Hauck‐Donner effect detected.

^b^ A natural‐log transformation was applied to serum PSA to remedy the right‐skewed distribution of the biomarker.

** Significant at *P* < .001.

* Significant at *P* < .05.

More aggressive PC was generally associated with much higher likelihood of treatment across all age groups. Prostate cancer survivors with more aggressive disease were more than twice as likely to receive surgery only (OR = 2.39; 95% CI 2.24‐2.54), more than 14 times as likely (OR = 14.55, 95% CI 12.57‐16.83) to receive surgery and radiation only, and more than 4 times as likely (OR = 4.15; 95% CI 3.9‐4.4) to receive combined therapies vs no definitive treatment compared to men with less aggressive disease (Table 2).

Non‐Hispanic black men were significantly less likely than any other racial/ethnic group to receive any cancer treatment modality: 47% less likely to receive surgery (OR = 0.53; 95% CI 0.48‐0.57; *P* < .001); 9% less likely to receive radiation only (OR = 0.91; 95% CI 0.83‐0.99; *P* < .05); 37% less likely receive surgery and radiation only (OR = 0.63; 95% CI 0.51‐0.77; *P* < .001); and 22% less likely (OR = 0.78; 95% CI 0.71‐0.86; *P*<.05) to receive combined therapies compared to NH white survivors (Table 2).

### 3.3 Impact of treatment received on PC–specific mortality

**FIGURE 1:**
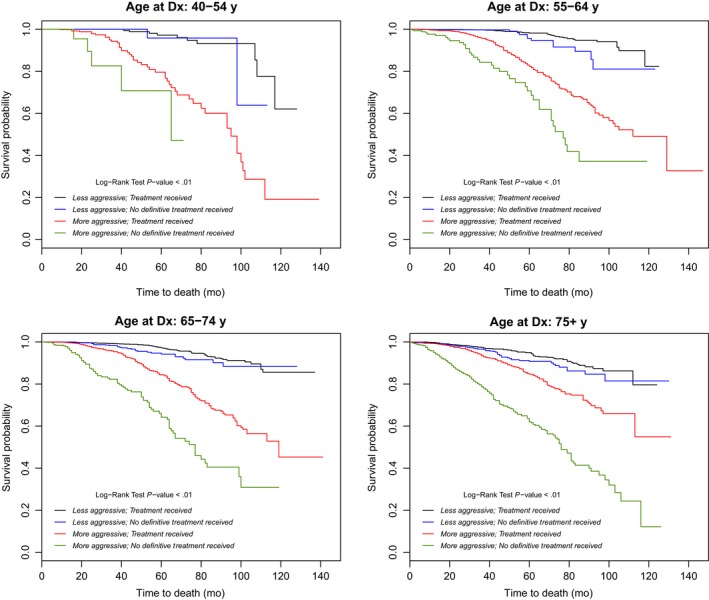
Trends in prostate‐cancer mortality based on treatment received and disease aggressiveness, by age at diagnosis (Dx). Kaplan‐Meier curves visually illustrate prostate cancer aggressiveness and indication of prostate cancer treatment received. Less aggressive disease is represented by lines in black (with treatment received) and blue (with no definitive treatment received). More aggressive disease is represented by lines in red (with treatment received) and green (with no definitive treatment received). The four subplots summarize these trends by age‐related subgroups. All log‐rank test *P*‐values were <.01

Based on the clinically recognized role of disease aggressiveness in determining appropriate treatment, we used Kaplan‐Meier curves to visually compare PC aggressiveness and indication of PC treatment, summarized by age group (Figure 1). For all age groups, the survival patterns remained similar; those diagnosed with more aggressive PC were more likely to die of PC compared to those with less aggressive disease (those receiving no definitive treatment experienced the highest mortality risk). Certain treatments appeared less beneficial for survival when administered to less aggressive diagnoses. Noting these patterns, a PH Cox regression model was fit to examine the effects of the interaction between treatment and aggressiveness, while adjusting for the other covariates that have been considered previously (Table 3).

**TABLE 3:** Stratified cox regressions by age group, reporting adjusted prostate cancer‐specific mortality risk

Age at Dx (y) stratified cox regressions	40‐54	55‐64	65‐74	75+
Treatment indicator				
No definitive treatment received (REF)	—	—	—	—
Treatment received^a^	0.77 (0.16, 3.67)	0.54 (0.29, 1.01)	0.65 (0.42, 0.99)*	0.69 (0.48, 0.99)*
Aggressiveness				
Less aggressive^^^ (REF)	—	—	—	—
More aggressive^#^	9.47 (1.41, 63.84)*	6.38 (3.32, 12.25)**	5.96 (3.84, 9.25)**	4.65 (3.42, 6.33)**
*Smoking* *^A^*	0.94 (0.85, 1.04)	0.98 (0.93, 1.03)	1.02 (0.98, 1.06)	1.01 (0.98, 1.05)
*Obesity* *^B^*	1.03 (0.95, 1.12)	1.02 (0.98, 1.06)	1.05 (1.02, 1.08)*	1.02 (1.00, 1.05)
*Physical inactivity* *^C^*	0.93 (0.81, 1.07)	0.99 (0.93, 1.05)	1.00 (0.96, 1.05)	1.00 (0.96, 1.04)
*Chronic drinking* *^D^*	0.80 (0.63, 1.03)	1.00 (0.90, 1.11)	0.95 (0.88, 1.03)	1.03 (0.95, 1.11)
*Daily fruits/vegetables* *^E^*	0.97 (0.87, 1.09)	1.00 (0.95, 1.05)	1.01 (0.97, 1.05)	0.96 (0.93, 1.00)*
Treatment × aggressiveness interaction				
Treatment received vs no definitive treatment received: more aggressive vs less aggressive	0.59 (0.08, 4.55)	0.96 (0.46, 2.01)	0.75 (0.45, 1.24)	0.52 (0.34, 0.80)*
*More aggressive*				
No definitive treatment received (REF)	—	—	—	—
Treatment received^a^	0.45 (0.13, 1.59)	0.52 (0.35, 0.78)*	0.49 (0.37, 0.63)**	0.36 (0.29, 0.45)**
*Less aggressive*				
No definitive treatment received (REF)	—	—	—	—
Treatment received^a^	0.77 (0.16, 3.67)	0.54 (0.29, 1.01)	0.65 (0.42, 0.99)*	0.69 (0.48, 0.99)*

Note

Cases with ‘Unknown’ or missing values for any of the covariates or the response were removed (n = 29 616 removed).

Main effects model with interaction: Treatment indicator + Disease aggressiveness + Race/ethnicity + Insurance status + Lymph node status + Serum PSA + Rurality + County‐level risk factors + Treatment indicator × Disease aggressiveness interaction.

County‐level (BRFSS region) risk factors we reassigned to PCR prostate cancer cases by county at the time of diagnosis, where counties within the same BRFSS region were linked with the same prevalence estimates as the region.

Hazard ratio (95% confidence interval).

Abbreviation: BRFSS, behavioral risk factor surveillance system; HT, hormone therapy; PCR, Pennsylvania Cancer Registry; REF, referent group.

^a^ Treatment received refers to any indication of the following modalities/combination of modalities: Surgery only, Radiation only, Surgery and radiation only, Radiation and/or surgery with adjuvant therapy (HT and/or chemo) treatment; those who received Adjuvant therapy only, or some other combination treatment therapy not listed, are categorized as ‘No treatment received’ for the purpose of these analyses.

** Significant at *P* < .001.

* Significant at *P* < .05.

Interpreting the interaction of aggressiveness with treatment status, the treatment effect only seemed to differ between aggressiveness groups for men ≥75 years. For other age groups, the treatment effect did not significantly differ by aggressiveness, and similar survival patterns among treatment indication could be observed in both aggressiveness groups. Generally speaking, in PCS 55‐74 years, those with more aggressive PC who received treatment had significantly lower risk of PC‐specific mortality than those who did not receive any definitive treatment. Furthermore, for men with more aggressive cancer who received treatment, those 55‐64 years were 48% less likely (HR = 0.52; 95% 0.35‐0.78); men 65‐74 years were 50% less likely (HR = 0.49; 95% CI 0.37‐0.63); and men 75 + years were 64% less likely (HR = 0.36; 95% CI 0.29‐0.45) to die from PC after receiving any treatment vs no definitive treatment. For men with less aggressive disease, PC survival benefits of treatment vs no definitive treatment were less universal. Older men 65‐74 years (HR = 0.65; 95% CI 0.42‐0.99) and ≥75 years (HR = 0.69; 95% CI 0.48‐0.99), with less aggressive disease were significantly more likely to benefit from treatment vs no definitive treatment (Table 3).

### 3.4 Role of environmental/behavioral risk factors on treatment recommendations and mortality

We observed some significant factors for receipt of treatment by reported lifestyle behaviors at time of diagnosis. Specifically, PCS who lived in counties with high prevalence of smoking were 5% less likely to get surgery only (OR = 0.95; 95% CI 0.94‐0.96); 4% less likely to receive radiation only (OR = 0.96; 95% CI 0.95‐0.97); 5% less likely to get surgery and radiation only (OR = 0.95; 95% CI 0.93‐0.98); and 7% less likely to get combined therapies vs no definitive treatment compared to men that live in counties with a lower percentage of smoking (Table 2). Prostate cancer survivors who lived in counties with high prevalence of obesity were also 4% less likely to receive surgery only (OR = 0.96; 95% CI 0.96‐0.97), 4% less likely to receive radiation only (OR = 0.96; 95% CI 0.95‐0.97), 8% less likely to receive surgery and radiation only (OR = 0.92; 95% CI 0.9‐0.94), and 3% less likely to receive combined therapies (OR = 0.97; 95%CI 0.96‐0.98) vs no definitive treatment compared men who live in counties with lower prevalence of obesity.

Interestingly, PCS who lived in counties with high rates of physical inactivity and had high rates of sufficient FV consumption were slightly *more* likely to receive cancer treatment vs no definitive treatment compared to men who lived in counties with high rates of physical activity and lower FV consumption. Men from these physically inactive counties were as follows: 2% more likely to have surgery only (OR = 1.02; 95% CI 1.01‐1.04), 2% more likely to have radiation only (OR = 1.02; 95% CI 1‐1.03), 4% more likely to get surgery and radiation only (OR = 1.04; 95% CI 1.01‐1.08), and 7% more likely to get combined therapies (OR = 1.07; 95% CI 1.05‐1.08) vs no definitive treatment compared to men living in counties with higher physical activity rates. Men living in counties with high prevalence of sufficient FV intake were 3% more likely to get surgery only (OR = 1.03; 95% CI 1.02‐1.04), 4% more likely to receive surgery and radiation only (OR = 1.04; 95% CI 1.02‐1.07), and 2% more likely to receive combined therapies (OR = 1.02; 95% CI 1.01‐1.03) vs no definitive treatment compared to men living in counties with lower rates of sufficient FV intake. Chronic alcohol use did not have a significant association with treatment recommendations.

Most environmental/behavioral risk factors did not have a significant impact on mortality. However, survivors 65‐74 years who lived in counties with high prevalence of obesity at diagnosis had a 5% increased risk of PC death (HR = 1.05; 95% CI 1.02‐1.08) compared to men who lived in counties with lower rates of obesity. Additionally, men ≥75 years who lived in counties with high FV consumption had a 4% reduction in PC mortality risk (HR = 0.96; 95% CI 0.93‐1.00) compared to men who lived in counties with lower prevalence of sufficient FV consumption.

## 4 DISCUSSION

We observed a general trend that suggests a decline in receipt of PC treatment associated with older age; more PCS diagnosed at ≥75 years were diagnosed with aggressive disease than men of other ages yet were less likely to receive any type of cancer treatment. Furthermore, compared to men 40‐54 years, PCS ≥ 75 years had much greater PC survival benefits from treatment, especially when diagnosed with more aggressive PC. Given the lack of consensus on optimal treatment approaches for PCS ≥ 75 years, this is a clinically significant finding.

While current guidelines prioritize severity of disease and life expectancy in guiding treatment decisions for men with localized PC,^25^, ^26^ Prostate cancer treatment has known problematic side effects, ranging from physical function deficits^27^ to increased risk of cardiovascular and metabolic health complications.^28^ The oldest survivors (especially those ≥75 years) may need special consideration in developing cancer treatment plans, even if they present with aggressive disease. Cancer‐specific geriatric assessment^29^ may be an invaluable tool in assessing individual disease experiences and ability to tolerate or benefit from more extensive treatment. More data on co‐morbidities and functional capacity are required to assess tolerability of systemic treatments and impact on longevity and quality of life.^30^

Likewise, younger men (eg, men diagnosed under age 65) should be fully informed about the risk of common cancer treatments, as they may expect to live for years or decades beyond diagnosis and treatment. Treatment side effects like incontinence and decreased sexual function are not life threatening, but can have psychological consequences^13^ and significantly reduce the quality of life for men, especially in their 40s or 50s. Non‐Hispanic black men in our study were also less likely to receive cancer treatment. These results are also supported in other studies that identified treatment and survival disparities for black men diagnosed with PC in the US.^31^, ^32^ In Pennsylvania, community facilities may lack cancer screening or cancer education programs, which may contribute to delays in early diagnosis/treatment and survival.^9^ Compared to other studies using state cancer registry data, such as Florida, PC disparities by age and race are not uncommon, or are disparities by rurality or geographic isolation.^33^ These kinds of rural‐urban disparities are often overlooked but require further investigation.^34^ Age at diagnosis and sociodemographic disparities are important factors,^6^ but must be considered in the context of an array of other relevant factors, including the health environment of the survivor.

Our study extends the literature by including county‐level behavioral risk factors as upstream predictors of PC risk and outcomes. Behavioral risk factor surveillance system regions with higher prevalence of obesity and smoking history stood out as unhealthy characteristics of these geographic areas that were negatively associated with most treatment modalities received in most age groups in our analysis. The American Society of Clinical Oncology's statement on obesity and cancer specifically addresses this matter,^35^ describing the role of obesity as a fuel that increases cancer risk, but also acts as an impediment to a favorable prognosis and effective delivery of systemic treatments. It is conceivable that treating physicians would weigh this risk in their treatment recommendations for PCS with excess weight or obesity. Similarly, cigarette smoking is an established predictor of increased mortality and cardiovascular disease risk in older men and PCS.^36^ As known cancer risk factors, the finding that individuals living in counties with high rates of these unhealthy behaviors may have received different cancer treatment advice is not unexpected, but still important to recognize and pursue as a future research question.

An unexpected finding among environmental/behavioral risk factors was that individuals diagnosed in the BRFSS regions with higher prevalence of physical *inactivity* and sufficient FV intake were slightly more likely to receive certain treatments. However, inactive individuals may also have increased risk of cardiovascular disease and metabolic changes,^37^ and that association may not be as well known in the medical community. These health behaviors might serve as apt targets for future research in cancer prevention and control.

Our study was strong in many ways. We used a robust, statewide cancer registry to identify a large sample of PCS and used county‐level surveillance data to contextualize the health environment of the PCS at the time of diagnosis. We examined the role of age at diagnosis using age‐related subgroups to understand treatment patterns, with a special focus on men ≥75 years, for whom there is little data to inform treatment recommendations and predict survival benefits.

Despite the richness of cases extracted from PCR, we faced challenges in classifying and analyzing registry records. For example, data recorded for more than one indicator were not always confirmatory and were sometimes contradictory. This was especially true for GS patterns, in which the secondary GS did not always match the total GS recorded. Certain assumptions had to be made to meet specific categorization criteria. However, this is a common challenge in using registry data for research purposes and we made every effort to document and compare related data to make the most robust definitions possible. County‐level estimates also provide context, but not precise estimates, of individual behaviors affecting PC prognosis and survival. More research connecting individual risk factors to PC outcomes would be a useful future research question.

Prostate cancer survivorship in the US is a growing phenomenon. As the consequence of a PC diagnosis has evolved, from a potentially life‐threatening diagnosis to a chronic disease diagnosis that requires long‐term management, opportunities to intervene to prevent symptoms and recurrence plus promote quality of years has taken shape. Given that PC is diagnosed at much older ages in most men, the importance of including men over age 75 years in clinical studies has never been more relevant.^38^ Lifestyle interventions, including exercise and strength training, may reduce cancer‐related symptoms and recurrence risk,^38^ especially in regaining or preserving function after completing hormonal therapy, surgery, or other treatments.

## CONFLICT OF INTEREST

The authors declare no potential conflicts of interest.

## AUTHOR CONTRIBUTIONS

S.M.B. led the conceptualization, design, analysis, and writing of this manuscript. M.W. led and E.W. and C.C. assisted with statistical analysis and data management for the study. N.Z. and R.H. provided clinical interpretation of data. R.H. provided senior leadership on the study. A.M. assisted in interpreting data and writing the manuscript. All co‐authors consented to submission of this manuscript.

## Data Availability

This study used existing data from the Pennsylvania Department of Health, and can be accessed based upon request and approval from the Pennsylvania Department of Health.
